# Acute Stress Response Profiles in Health Workers Facing SARS-CoV-2

**DOI:** 10.3389/fpsyg.2021.660156

**Published:** 2021-05-31

**Authors:** Luca Moderato, Davide Lazzeroni, Annalisa Oppo, Francesco Dell’Orco, Paolo Moderato, Giovambattista Presti

**Affiliations:** ^1^Guglielmo da Saliceto Hospital, Piacenza, Italy; ^2^IRCCS Fondazione Don Gnocchi, Florence, Italy; ^3^Department of Psychology, Sigmund Freud University, Milan, Italy; ^4^Istituto Europeo per lo Studio del Comportamento Umano, ONLUS, Parma, Italy; ^5^Department BLEC, Libera Università di Lingue e Comunicazione IULM, Milan, Italy; ^6^Department of Human and Social Sciences, Kore University of Enna, Enna, Italy

**Keywords:** SARS-CoV-2, PTSD profiles, distress symptoms, health workers, descriptive survey study, COVID-19, Italy, lockdown

## Abstract

**Objective:**

The study is an explorative investigation aimed to assess the differences in acute stress response patterns of health workers facing coronavirus disease 2019 (COVID-19) during Italy’s first lockdown.

**Methods:**

A cross-sectional investigation using convenience sampling method was conducted in Italy during April 2020. Eight hundred fifty-eight health workers participated in the research filling out self-report measures including Patient Health Questionnaire (PHQ-9), Generalized Anxiety Disorder (GAD-7), Insomnia Severity Index (ISI), and Impact of Event Scale–Revised (IES-R).

**Results:**

Moderate/severe depression was found in 28.9% (95% CI, 25.8–32.04), moderate/severe anxiety in 55.4% (95% CI, 51.9–58.8), insomnia in 15% (95% CI, 12.5–17.5), and distress in 52.5% (95% CI, 48.5%–56.6) of participants. The 3% of health workers reported frequent suicidal thoughts. Female sex, working for >15 h/week in a COVID-19 unit, and living apart from family were associated with a significantly higher risk of distress, anxiety, insomnia, depression, and functional impairment. Four profiles were identified on the basis of psychopathological measures: Profile_0 included 44% (*N* = 270); Profile_1, 25.6% (*N* = 157); Profile_2, 19.1% (*N* = 117); and Profile_3, 11.3% (*N* = 69) of participants. Results showed a significant effect for Profiles X IES-R (η^2^ = 0.079; *f* = 0.29), indicating that in all profiles, except for Profile_0, avoidance scale is lower than hyperarousal and intrusion symptoms scales of the IES-R. This characteristic could be a probable index of the control exerted by the responders to not fly away from their job.

**Conclusion:**

The identification of specific profiles could help psychiatrists and emergency psychologists to build specific interventions in terms of both primary and secondary prevention to face future waves of the COVID-19 outbreak.

## Introduction

Italy was the second country to be strongly hit by severe acute respiratory syndrome coronavirus 2 (SARS-CoV-2) outbreak. The first case was diagnosed on February 21, 2020, and national lockdown was declared on the March 8. At the end of the first wave of the outbreak, the number of deaths was approximately 35,000 with 250,000 people infected (COVID-19 Situazione Italia, 2020). An unknown respiratory disease, for which no specific cure is still available, engenders a big challenge for health workers. In addition, several critical factors might undermine the capacity of health workers to treat patients, including insufficient number of Intensive Care Unit (ICU) beds available related to the overwhelming number of patients that are hospitalized simultaneously, the disruption of daily medical routine, and need of making life-and-death decisions.

Recently, researchers have called for a focus toward the mental health needs of frontline healthcare workers (Zaka et al., 2020). There is a strong evidence that acute stress response (ASR) risk increases during natural disasters, for healthcare professionals, and nurses among them (Demartini et al., 2020; Xiaorong et al., 2020). These data are consistent with previous studies conducted at the time of 2003 severe acute respiratory syndrome (SARS) epidemic, which showed that mental health problems were present in 57% of healthcare workers (Tam et al., 2004).

Early evidence suggests that the COVID-19 outbreak may cause clinically relevant adverse psychological reactions, potentially stronger than SARS in 2003 (Brooks et al., 2020). Psychological distress, anxiety (da Silva et al., 2020), and depressive symptoms have been reported in various health worker groups in China (Kang et al., 2020; Lai et al., 2020; Lv et al., 2020; Zhu J. et al., 2020), Jordan (Naser et al., 2020), and Italy (Demartini et al., 2020; Rossi et al., 2020). Rogers et al. (2020) highlighted that the risk of mental illness of infected heath workers during life-threatening pandemics is about fourfold the infected general population. In addition, the intensity of caring, the donning of personal protective equipment, and purposely staying away from home, to avoid infecting the loved ones, may amplify feelings of social isolation, which is known as an important resilience factor (Lv et al., 2020).

Trauma and related disorders are often an unseen pandemic that follows the viral ones, whose risk factors are routed in the distress experienced. Recent data on Chinese general population highlighted that 14% of young subjects was affected by PTSD symptoms, mediated by negative coping (Liang et al., 2020).

As health workers are and will be exposed to unprecedented levels of intensive existential threat, an urgent and systematic psychological support is needed (COVID-19 Situazione Italia, 2020); in this context, hospitals should implement strategies to prevent, early detect, support, and treat work-related stress (Paladino et al., 2017; Schenk et al., 2017).

The aim of this study was to assess the type and magnitude of ASR risks related to exposure to COVID-19 on Italian health workers and investigate whether specific profiles, defined according to the allostatic load, might address prevention strategies of further mental disorders or *ad hoc* therapies.

## Materials and Methods

### Study Design

The study is a web-based cross-sectional study conducted between April 15 and 28, 2020, when confirmed COVID cases exceeded 100,000 (COVID-19 Situazione Italia, 2020). To compare the mental health outcomes, samples were stratified both by their geographic location (i.e., north west, north east, center, and south) and by emergency area (Red vs. No-Red Zone). The Red Zone includes the Lombardy region and 14 additional provinces. Because of the web-based snowball sampling strategy, response rate could not be calculated.

### Participants

Lockdown measures prevented direct contact with such a population. Participants were recruited with a convenience sampling method through social media, messages, emails, and especially through Facebook closed groups of Italian health workers.

### Assessment

Online-based questionnaire was divided in two parts: the first investigated demographic characteristics, depression and anxiety symptoms, insomnia and functional impairment, and distress the second.

Demographic information included the following: occupation (physician, nurse, others), sex (male, female), age (years); geographic location (the Italian province where the respondent worked); living at distance from significant ones, or with partner/family, friends; hours of weekly exposure to COVID patients (1–15 h, 16–30 h, 31–45 h, or >45 h); work setting (frontline vs. second line); and the availability of personnel protective equipment (PPE; yes, no). Participants were also asked to estimate if COVID patients were increasing, decreasing, or stable in their region; finally, they were asked to report if the subject or partner or a family member was positive, negative, or not tested for COVID-19.

Furthermore, this survey included self-reported measures.

Depressive symptoms were assessed with Patient Health Questionnaire (Spitzer, 1999). The Patient Health Questionnaire (PHQ-9) is a nine-item measure that has a total score ranging from 0 to 27, with higher scores indicating depressive problems. The validation study showed a good agreement between PHQ diagnoses and those of independent mental health professionals. Furthermore, in a meta-analysis including 14 studies, a pooled sensitivity of 0.80 (95% CI, 0.71–0.87) and a pooled specificity of 0.92 (95% CI 0.88–0.95) were found (Gilbody et al., 2007). In this sample, Cronbach’s alpha was 0.848.

Anxiety and distress symptoms were assessed with Generalized Anxiety Disorder 7-item (GAD-7) scale (Spitzer et al., 2006). The total score ranges from 0 to 21, with higher scores indicating higher anxiety and distress symptoms. The GAD-7 is a tool with excellent psychometric properties and a useful tool for detecting GAD and assessing its severity both in clinical practice and for research purpose. The internal consistency of the GAD-7 was excellent (Cronbach’s alpha = 0.92). In this sample, Cronbach’s alpha was 0.911.

Quality of sleep and Insomnia were assessed with the Insomnia Severity Index (Morin, 1993). The Insomnia Severity Index (ISI) is a seven-item measure that has a total score ranging from 0 to 28, with higher scores indicating sleep problems. The ISI is an easily administrable self-reported instrument that can be used both for screening and for evaluation of treatment and shows good psychometric properties. In this sample, Cronbach’s alpha was 0.779.

Distress caused by a potential traumatic event was assessed with the Impact of Event Scale–Revised (Weiss, 2007). The Impact of Event Scale–Revised (IES-R) is a 22-item measure that has a total score ranging from 0 to 88 and three subscales that evaluate avoidance, hyperarousal, and intrusion symptoms calculated with items’ mean (value ranges from 0 to 4). Higher scores, both in the total score and in the subscales, correspond to more severe conditions. The IES-R showed very good psychometric properties: high levels of internal consistency (Cronbach’s alpha around 0.95) and discriminative validity have been reported. In this sample, Cronbach’s alpha was 0.934.

Cutoffs were, respectively, PHQ-9 ≥ 15 (Spitzer, 1999), GAD-7 ≥ 10 (Gilbody et al., 2007), ISI ≥ 15 (Spitzer et al., 2006), and IES-R ≥ 33 (Weiss, 2007).

### Severity Symptom Index and Stress Response

The rationale that guided this choice was not determined by a specific symptomatology (i.e., anxiety, depression, insomnia, and all possible combinations between these symptoms); rather, a quantitative criterion was chosen that is consistent with the perspective of allostatic load. Thus, positivity or negativity to PHQ-9, GAD-7, and ISI clinical cutoffs was used to identify four severity symptoms profiles: all indexes negative (Profile_0), equal or above cutoffs either in one (Profile_1), two (Profile_2), or all of the three scales (Profile_3). We evaluated how overall IES-R distress and its three components are distributed over those profiles.

### Statistical Analysis

Categorical data are presented as n (%) and continuous data as means (±SD). A 95% confidence interval (CI) indicates uncertainty around the estimates. Chi-square was used to evaluate differences between categorical variables, whereas independent *t* tests and one-way ANOVA were used when appropriate to investigate differences between continuous variables. To determine potential risk factors for symptoms of depression, anxiety, insomnia, and distress in participants, logistic regression analysis was performed, and the associations between risk factors and outcomes are presented as odds ratios (ORs) and 95% CIs. A 4 × 3 mixed analysis of variance model tested the differences in the IES-R subscales in the four identified profiles. *Post hoc* tests were adjusted for multiple comparisons using Bonferroni correction. To clarify the magnitude of the effect size, η^2^ was rescaled in *f* index. Effect size is defined as small, medium, or large, based on *f* equal to 0.1, 0.25, and 0.40, respectively (Cohen, 1988). SPSS software version 21.0 (IBM Corp.) was used for statistical analysis, and the significance level was set at α = 0.05, and all tests were two-tailed.

### Ethics

The study adhered to all ethical principles for the good conduct of research with humans outlined by the Declaration of Helsinki. The study was approved by the Institutional Ethical Committee of the Department of Human and Social Science of the Kore University of Enna, with respect to scientific content and compliance with the Italian applicable research and human subjects’ regulations (protocol number: UKE-IRBPSY-05.20.01). Online informed consent was provided by all survey participants prior to their enrollment. Participants could terminate the survey at any time they desired. The survey was anonymous, and confidentiality of information was assured.

### Data Sharing

Deidentified participant data will be shared as required from July 1, 2020 by the policy of the Journal. Data will be available as a spreadsheet with clear labels.

## Results

### Characteristics of the Study Population

Eight hundred fifty-eight participants flagged the informed consent and completed the first part of the online survey. Table 1 reports demographic characteristics, and Table 2 reports workplace setting of the participants by sex and emergency area (Red Zone vs. No-Red Zone). Overall, mean age of participants was 41.25 (*SD* = 10.14, *range* = 22–72), the large majority being female (84.4%), physicians (76.7%), and married or cohabiting (75.8%), and 21.8% of them chose to live isolated. Frontline workers were 50.1%. Weekly exposure time in COVID setting was 1–15 h for 25.6%, 16–30 h for 12.5%, 30–45 h for 25.9%, and more than 45 h for 8% of respondents, while 238 data were missing; 65% of participants worked in the Red Zone (Table 2). Overall, 62.4% declared that PPEs were available. Respondents declared that PPE availability was about five times higher in frontline than in second line settings (OR = 5.12; 95% CI, 3.74–7.09); however, 26.1% of frontline staff considered it inadequate.

**TABLE 1:** Characteristics of study population.

	Overall (*N* = 858)		Male (*N* = 132)		Female (*N* = 724)		Red Zone (*N* = 430)		No-Red Zone (*N* = 416)	
Age Mean (SD)	41.25	10.14	40.20	10.77	41.47	10.07	39.7	9.65	42.8	10.35
**Sex**										
Female N (%)	724	84.4%	–	–			350	81.4%	364	87.5%
Male N (%)	132	15.4%	–	–			79	18.4%	51	12.3%
Missing N (%)	2	0.2%	–	–			1	0.2	1	0.2%
**Region**										
North-West	255	29.7%	38	28.8%	217	30.0%	182	42.3%	73	17.5%
North-East	343	40.0%	65	49.2%	277	38.2%	244	56.7%	99	23.8%
Center	110	12.8%	8	6.0%	102	14.0%	4	1%	106	25.5%
South	138	16.1%	19	14.5%	118	16.4%	–	–	138	33.2%
Missing data	12	1.4%	2	1.5%	10	1.4%	–	–	–	–
**Red Zone**										
Yes	430	50.1%	79	59.8%	350	48.3%	–	–	–	–
No	416	48.5%	51	38.6%	364	50.3%	–	–	–	–
Missing data	12	1.4%	2	1.6%	10	1.4%				
**Occupation**										
Physician	658	76.7%	108	81.8%	550	76%	314	73.0%	332	79.8%
Nurse	149	17.4%	18	13.6%	131	18.1%	84	19.5%	65	15.7%
Other	49	5.7%	6	4.6%	43	5.9%	31	7.3%	18	4.3%
Missing	2	0.2%	–	.			1	0.2%	1	0.2%
**Housing situation**										
Alone	161	18.7%	22	16.7%	139	19.2%	79	18.4%	81	19.5%
Roommates	35	4.1%	5	3.8%	30	4.1%	15	3.5%	18	4.3%
Married/cohabiting	508	59.2%	82	62.1%	426	58.7%	269	62.6%	233	56.0%
Married/cohabiting but Isolated	142	16.5%	20	15.2%	122	16.8%	64	14.9%	77	18.5%
Missing data	12	1.5%	3	2.2%	9	1.2%	3	0.6%	7	1.7%
**Infected COVID-19**										
Yes	71	8.3%	7	5.3%	64	8.8%	46	10.7%	23	5.5%
No	438	51.1%	77	58.3%	300	41.4%	178	41.4%	230	55.3%
Not tested	346	40.3%	46	34.8%	359	49.7%	206	47.9%	162	38.5%
Missing data	3	0.3%	7	5.3%	1	0.1%	–	–	1	0.2%
**Partner infected COVID-19**										
Yes	34	4.0%	4	3.0%	30	4.1%	20	4.7%	14	3.4%
No	399	46.5%	76	57.7%	329	45.4%	189	44.0%	205	49.3%
Not tested	200	46.6%	46	34.8%	343	47.5%	216	50.2%	179	43.0%
Missing data	25	2.9%	2	1.5%	22	3.0%	5	1.2%	18	4.3%
**Family member infected COVID-19**										
Yes	62	7.2%	8	6.1%	54	7.5%	41	9.5%	19	4.6%
No	491	57.2%	76	57.6%	413	57.0%	234	54.4%	252	60.6%
Not tested	300	35.0%	46	34.8%	254	35.1%	154	35.8%	143	34.4%
Missing data	5	0.6%	2	1.5%	3	0.4%	1	0.2%	2	0.5%

**TABLE 2:** Workplace setting of the study population.

	Overall (*N* = 858)		Male (*N* = 132)		Female (*N* = 724)		Red Zone (*N* = 430)		No-Red Zone (*N* = 416)	
**Workplace unit**										
Intensive Care Unit	114	13.3%	38	28.8%	96	13.3%	52	12.1%	61	14.7%
Clinical Setting	340	39.6%	65	49.2%	288	39.6%	201	46.7%	136	32.7%
Emergency Department	73	8.5%	8	14.4%	63	8.7%	25	5.8%	46	11.1%
Diagnostic Care Unit	62	7.2%	19	14.4%	122	16.9%	30	7.1%	31	7.4%
Out-of-hospital	141	16.4%	2	1.5%	52	7.2%	65	15.1%	74	17.8%
Other	128	14.9%	79	59.8%	103	14.2	57	13.3%	68	16.3%
Missing data	–	–	2	1.5%	–	.	–	–	–	–
**Work setting**										
Frontline	598	69.7%	98	74.2%	500	69.1%	346	80.5%	246	59.1%
Second-line	256	29.8%	33	25.0%	223	30.8%	83	19.3%	169	40.7%
Missing data	4	0.5%	1	0.8%	1	0.1%	1	0.2%	1	0.2%
**PPE availability**										
Yes	533	62.1%	88	66.7%	445	61.5%	307	71.4%	220	52.9%
No	325	37.9%	44	33.3%	279	38.5%	123	28.6%	196	47.1%
**Exposure to COVID-19**										
1–15 h	220	25.6%	37	28.9%	183	25.3%	111	25.8%	107	40.8%
16–30 h	107	12.5%	13	9.8%	94	13.0%	62	14.4%	43	16.4%
31–45	222	25.9%	39	29.5%	183	25.3%	127	29.5%	93	35.5%
>45 h	71	8%	12	9.1%	59	8.1%	51	11.9%	19	7.3%
Missing data	238	27.7%	31	23.5%	205	28.3%	79	18.4%	154	37.0%

*PPE, personal protective equipment.*

Six hundred thirteen (71.4%) participants completed the second part of the survey, which included the IES-R. Participants who did not complete the survey (*N* = 245) were significantly older than those who did [42.7 ± 10.5 vs. 40.7 ± 10.0; *t*(856) = 2.73; *p* < 0.001], with a very small effect size (*d* = 0.20). No significant differences were observed in other sociodemographic and clinical characteristics.

### Main Outcome Measures and Associated Factors

**FIGURE 1:**
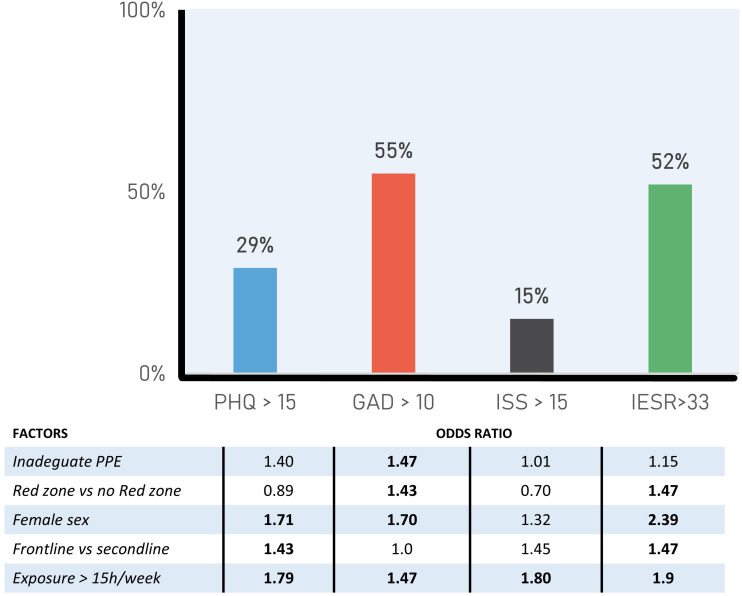
Prevalence depression, anxiety, insomnia, work impairment, and distress. Above factors with relative odds ratio are indicated. PHQ-9, Patient Health Questionnaire; GAD-7, Generalized Anxiety Disorder; ISI, Insomnia Severity Index; IES-R, Impact of Event Scale–Revised.

Overall, 28.9% (95% CI, 25.8–32.04) of participants reported moderate to severe depression, 55.4% moderate or severe anxiety (95% CI, 51.9–58.8), 15.0% insomnia (95% CI, 12.5–17.5), and 52.5% distress (95% CI, 48.5%–56.6) (Figure 1 and Supplementary Figure 1). Age was significantly associated with anxiety (*r* = −0.071; *p* = 0.032), with a very small effect size, unrelated with depression (*r* = −0.013; *p* = 0.699), insomnia (*r* = 0.013, *p* = 0.694), and distress (*r* = −0.026, *p* = 0.518). Table 3 reports the odds ratios with 95% confidence intervals of having a score above clinical (moderate to severe level) threshold of the main outcome measures as a function of demographic characteristics and workplace setting.

**TABLE 3:** Odds ratios for outcomes according to different covariates.

	PHQ-9 ≥ 15		GAD-7 ≥ 10		ISI ≥ 15		IES ≥ 33	
	OR	95% CI	OR	95% CI	OR	95% CI	OR	95% CI
**Sex**								
Female	**1.71**	**1.09**–**2.68**	**1.90**	**1.30**–**2.75**	1.32	0.75–2.31	**2.39**	**1.49**–**3.8**
Male	1		1		1		1	
**Red Zone**								
Yes	0.89	0.66–1.21	1		0.70	0.48–1.03	1	
No	1		**1.43**	**1.04**–**1.75**	1		**1.47**	**1.05**–**2.0**
**Occupation**								
Physician	0.99	0.69–1.40	0.96	0.67–1.38	1		0.82	0.54–1.23
Nurse	1		1		**1.85**	**1.18**–**2.85**	1	
**Housing situation**								
Alone	1.32	0.90–1.95	1.02	0.71–1.92	1.34	0.82–2.19	1.32	1.18–2.99
Roommates	1.13	0.52–2.38	1.45	0.71–2.94	1.15	0.43–3.07	0.84	0.38–1.84
Married/cohabiting but Isolated	**1.51**	**1.07**–**2.25**	1.31	0.90–1.92	**1.85**	**1.14**–**2.98**	**1.88**	**1.19**–**2.99**
Married/cohabiting	1		1		1		1	
**Infected COVID-19**								
Yes	**1.82**	**1.08**–**3.07**	1.26	0.76–2.05	1.54	0.81–2.93	**2.33**	**1.25**–**4.35**
Not tested	1.18	0.87–1.62	1.27	0.95–1.68	1.01	0.73–1.63	1.02	0.73–1.43
NO	1	1	1	1	1	1	1	1
**Partner infected COVID-19**								
Yes	0.99	0.5–2.19	1.06	0.53–2.13	0.64	0.19–2.16	1.03	0.44–2.46
Not tested	1.23	0.90–1.66	**1.32**	**1.0**–**1.74**	1.36	0.92–2.0	1.22	0.88–1.70
No	1		1		1		1	
**Family member infected COVID-19**								
Yes	**1.91**	**1.11**–**3.30**	1.29	0.75–2.20	**2.04**	**1.06**–**3.92**	1.06	0.58–1.95
Not tested	1.27	0.93–1.75	1.19	0.89–1.60	1.47	0.99–2.20	1.08	0.77–1.52
No	1		1		1		1	
**Work setting**								
Frontline	**1.43**	**1.05**–**1.95**	1.01	0.76–1.34	1.45	0.97–2.17	**1.47**	**1.06**–**2.05**
Second-line	1		1		1		1	
**PPE availability**								
No	1.01	0.81–1.50	**1.56**	**1.18**–**2.07**	1.01	0.88–1.14	1.15	0.83–1.60
Yes	1		1		1		1	
**Exposure to COVID-19**								
>15 h	**1.77**	**1.22**–**2.58**	1.28	0.92–1.78	**1.80**	**1.11**–**2.93**	**1.90**	**1.27**–**2.86**
<15 h	1		1		1		1	1

*Statistically significant ORs are in boldface. PHQ-9, Patient Health Questionnaire; GAD-7, Generalized Anxiety Disorder; ISI, Insomnia Severity Index; IES-R, Impact of Event Scale–Revised; PPE, personal protective equipment.*

Female participants show a greater likelihood of having moderate-to-severe depression (30.6 vs. 20.5%; OR = 1.71; 95% CI, 1.09–2.68; *p* = 0.02), anxiety (57.8 vs. 42%; OR = 1.90; 95% CI, 1.30–2.75; *p* = 0.001), and distress (55.7 vs. 34.4%; OR = 2.39; 95% CI, 1.49–3.38; *p* < 0.001) symptoms than male.

Being directly exposed to the danger of contagion is one of the main stressors. Specifically, working for >15 h/week in a COVID-19 unit is associated with higher risk of distress (59.9 vs. 44%; OR = 1.90; 95% CI, 1.27–2.86; *p* = 0.002), severe insomnia (18.8 vs. 11.4%; OR = 1.80; *p* = 0.018), and depression (34.8 vs. 23.2%; OR = 1.79; *p* = 0.003). However, prevalence of anxiety symptoms is similar in both COVID (>15 weekly hours) and no-COVID settings (59 vs. 53%; OR = 1.28; 95% CI, 0.92–1.78; *p* = 0.14).

In order to identify predictors significantly associated to main outcome measures, four stepwise logistic regression models adjusted by age and sex were fitted. The first shows that working >15 weekly hours inside a COVID-19 (OR = 1.80; 95% CI, 1.23–2.64) increases the probability of moderate/severe depression. Sex (OR = 1.97; 95% CI, 1.33–2.91), age (OR = 0.98; 95% CI, 0.96–0.99), and reporting inadequate PPE availability (OR = 1.73; 95% CI, 1.23–2.34) are positively correlated to increased anxiety. Older participants (OR = 1.024; 95% CI, 1.001–1.047) working >15 weekly hours in the COVID area (OR = 1.99; 95% CI, 1.20–3.27) had a greater likelihood to suffer from severe insomnia. Finally, a greater likelihood to report distress symptoms was also found in staff working >15 weekly hours in the COVID area (OR = 1.92; 95% CI, 1.27–2.92) and disclosing being positive to COVID-19 (OR = 2.51; 95% CI, 1.22–5.15).

### Profiles of Acute Stress Response per the Severity Symptom Index

Profile_0 included 44% (*N* = 270); Profile_1, 25.6% (*N* = 157); Profile_2, 19.1% (*N* = 117); and Profile_3, 11.3% (*N* = 69) of participants. Partial correlations between the three subscales correct for profiles of the IES-R showed a moderate to great effect size that ranges between 0.540 and 0.720, while zero-order correlations showed a greater effect size that ranges between 0.655 and 0.825.

**FIGURE 2:**
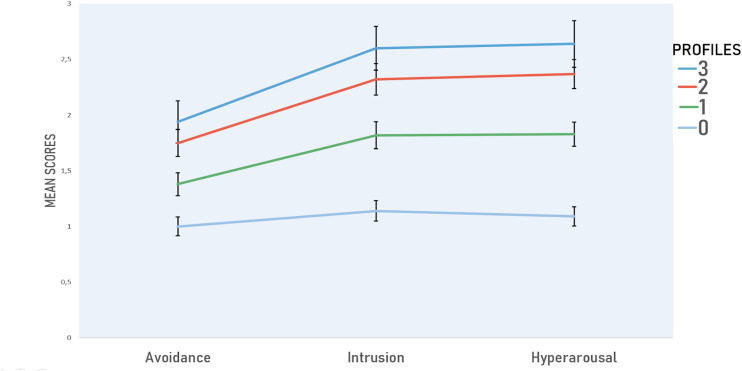
Profiles and relative scores of Avoidance, Intrusion, and Hyperarousal. Positivity or negativity to PHQ-9, GAD-7, and ISI clinical cutoffs were used to identify four severity symptoms profiles: all indexes negative (Profile_0), equal or above cutoffs either in one (Profile_1), two (Profile_2), or all of the three scales (Profile_3). PHQ-9, Patient Health Questionnaire; GAD-7, Generalized Anxiety Disorder; ISI, Insomnia Severity Index.

To better clarify how the subscales of the IES-R behave in the different profiles, an ANOVA (4 × 3) was performed. In particular, we are interested in the interaction effect because, when interaction effect is present, it means that interpretation of the main effects is incomplete. The ANOVA model showed a main effect of IES-R subscale [*F*(2,1214) = 166.57; *p* < 0.001; η^2^ = 0.215; *f* = 0.52], and a main effect of profiles [*F*(3,607) = 131.13; *p* < 0.001; η^2^ = 0.393; *f* = 0.80]. A significant effect for Profiles X IES-R was observed [*F*(6,1214) = 17.31; *p* < 0.001; η^2^ = 0.079; *f* = 0.29]. *Post hoc* analysis indicated that Profile_0 showed significantly lower scores in all IES-R subscales than Profile_1 (*p* < 0.001), Profile_2 (*p* < 0.001), and Profile_3 (*p* < 0.001) (Figure 2). Profile_1 showed significantly lower scores in all IES-R subscales than Profile_2 (*p* < 0.001) and Profile_3 (*p* < 0.001), while Profile_2 and Profile_3 were similar (*p* = 0.06).

Finally, in Profile_0, the partial eta-square was 0.032; in profile_1, 0.228; in profile_2, 0.345; and in the profile_3, 0.378.

## Discussion

Our data confirm that during the COVID-19 pandemic, as for Ebola Virus Disease (McMahon et al., 2016) and SARS (Verma et al., 2004), clinically relevant mental health symptoms can be identified in frontline and second-line healthcare staff. A higher percentage of participants with moderate to severe depression (28.9%), anxiety (55.4%), insomnia (15%), and distress symptoms (52.5%) than previously reported was found in our study (Demartini et al., 2020; Lai et al., 2020; Lv et al., 2020; Naser et al., 2020; Rossi et al., 2020; Wu et al., 2020; Zhu J. et al., 2020).

Comparison with published data highlights qualitative and quantitative differences in collection, participants, and clinical criteria chosen to group them. However, PHQ-9, GAD-7, ISI, and IES-R have been extensively used.

In other studies, 13–18% of Chinese (Lai et al., 2020; Lv et al., 2020; Zhu Z. et al., 2020), 21.3% of Jordan (Naser et al., 2020), and 24.7% of Italian staff (Rossi et al., 2020) had PHQ-9 scores ≥ 15, the latter being albeit lower but not so far from ours (29%). Half of our participants had GAD-7 scores >10, compared to 24.1% in Chinese (Zhu Z. et al., 2020) and 32.8% in Jordanian health workers (Naser et al., 2020). In the other Italian study, GAD-7 scores ≥ 15 were detected in 19.8 vs. 23.3% of our subjects (Rossi et al., 2020). Sleeping problems were reported in 15% of subjects compared to 7.8% reported by Lai et al. (2020). Only 2% of participants in this study show an ISI score ≥ 22 vs. 8.27% of another Italian study (Rossi et al., 2020). IES-R scores >33 were reported by 29.8% of respondents in one Chinese study (Zhu Z. et al., 2020) compared to 52% of our population. Lai et al. (2020) used different cutoffs and reported moderate (IES-R, 26–43) in 24.5% and severe (IES-R, 44–88) in 10.5% of subjects.

Overall, our data offer a different picture from previous reports and closer to the other Italian study published so far, given the greater number of days into the COVID-19 fight.

Study groups, hospitalization rates, workload, frontline vs. second-line staff, and period of data collection with respect to COVID-19 diffusion, as well as being the second country hit by the virus, with scarce knowledge on the disease at that time, can account for those differences. Our group, for example, shows higher proportion of frontline staff, 69.7 vs. 52.7% of the other Italian study down to a range from 41 to 34.3% of other Chinese studies (Kang et al., 2020; Lai et al., 2020; Lv et al., 2020; Zhu Z. et al., 2020). No student took part in this study, while in other published papers, the proportion was up to 75–85% (Kang et al., 2020; Lai et al., 2020; Zhu Z. et al., 2020). In some studies (Kang et al., 2020; Zhu Z. et al., 2020), resilience practices, e.g., access to material, Balint groups, or social networks, were investigated, and lower prevalence of mental health issues than ours can partially be a result of those practices. No data were collected on personal strategies for stress management in our participants. Gender differences have been reported in both trauma-related emotional avoidance and responding (Schick et al., 2020). Our data confirm that the risk of mental health disorders is higher in women (Lai et al., 2020; Rossi et al., 2020).

Length of exposure to stressors can influence ASR. During our data collection period, close to the end of the long pandemic wave, COVID-19 cases increased from 165,000 to 203,000 (COVID-19 Situazione Italia, 2020). Data from Chinese studies (Kang et al., 2020; Lai et al., 2020; Lv et al., 2020; Zhu Z. et al., 2020) were collected between end of January (WHO confirmed case *n* = 7736) and end of February 2020 (WHO confirmed case *n* = 142,823). To measure stress-related workload, participants were asked to self-report the number of weekly hours in COVID areas; no other research accounted for this variable.

Identification of at-risk groups and type of stress reactions is pivotal to prevention and treatment measures. We grouped into four severity symptoms profiles, in terms of allostatic load, and analyzed in relation to IES-R avoidance, hyperarousal, and intrusion subscales, an interesting pattern in data emerged: it is enough for either PHQ-9, GAD-7, and ISI to be in the clinical range for distress to rise. A closer look to means and SD of scales in Profile_1 (the mildest of the three) shows that they can even be slightly under threshold. Furthermore, in all profiles, except for Profile_0, avoidance scale is descending, a probable index of the control exerted by the responders to not fly away from their job. In addition, it is likely that the intrusive symptoms are a direct effect of hyperarousal, supported also by the fact that it was not possible to emit avoidance behavior.

Odds ratio analysis in our population suggests that ASR risks change along a COVID closeness-related uncertainty–certainty bipole. When uncertainty is higher (No-Red Zone, partner not tested for COVID, inadequate personal protective equipment availability), risk of detecting clinical anxiety levels is roughly 1.5 higher. Risks for depression rises when either the staff or partner is COVID+ or working in the frontline. Living away from significant ones elevates ORs in all indexes except for anxiety. More than 15 weekly hours of frontline work increases risks of all ASR symptoms. Reactions to COVID-19 follow a typical learned-helplessness two-phase pattern, going from anxiety when anticipating danger to depression when into it.

There are several limits to the present study: the snowballing sampling procedure may casually select a non-representative sample, and symptoms reporting may be exaggerated. Responders may amplify, misrepresent, or underreport symptoms in order to make their situation seems worse, different, or minimize their problems. Pre-COVID psychological status is unknown and might have an influence on symptoms reporting. There is no comparison group in the general population in the same geographical area that can help evaluate if the observed effects are specific to health workers or general, although using different psychological measures, high to very high depression and anxiety levels were found in 32.8 and 18.7% of 2766 Italian respondents to a questionnaire 1 month earlier than ours (Mazza et al., 2020). Moreover, although our results showed an association between inadequate PPE availability (OR = 1.73; 95% CI, 1.23–2.34) and anxiety, it cannot be excluded that the level of anxiety reported by health workers engaged in facing a pandemic may have partially distorted perceptions regarding protective equipment.

To our knowledge, no dedicated psychological support was available during the first days of the COVID-19 pandemic wave. By the way, this item was not investigated; therefore, we cannot exclude this bias.

Finally, this is a still picture that assesses heath workers’ psychological status in a specific moment in time, and a programmed follow-up phase will later help discriminate mental health trajectories.

Occupational stress is common in healthcare workers and can jeopardize not only their mental health but also the quality of their work (Steptoe and Kivimäki, 2013; Ruotsalainen et al., 2015). A healthy workplace is essential to maintain hospital services and could be cost effective from the employer’s perspective (Wijnen et al., 2020). Given the traumatic experiences of COVID-19 pandemic, other protective measures in addition to PPE are needed to maintain the healthcare staff’s biological and mental well-being (Lv et al., 2020). COVID-19 pandemic has underlined the need for evidence-supported interventions to enhance psychological flexibility (Gloster et al., 2020; Presti et al., 2020).

To date, data from many EU countries show that the pandemic is far from being under control. Due to summer activities (travels, crowded beaches, restaurants, disco, etc.), the cases of infection are increasing and the number of hospital admissions, too. This is true in Spain, in Germany, in France, and in Italy as well. We are entering the phase that Tomas Pueyo called the Dance: the first phase, the Hammer, i.e., the lockdown, was needed to gain time; the second, the Dance, is aimed to live with the epidemic while waiting for the availability of a vaccine (Pueyo, 2020).

In the Dance time, schools are opening, and the first effects in the EU countries are worrying; to dance with the outbreak, we must, among other things, monitor in real time what is happening in the hospitals and be ready to support the health workers psychologically and physically.

## Data Availability Statement

The raw data supporting the conclusions of this article will be made available by the authors, without undue reservation.

## Ethics Statement

The studies involving human participants were reviewed and approved by the IRB Department of Human and Social Sciences – Kore University of Enna. The patients/participants provided their written informed consent to participate in this study.

## Author Contributions

All authors listed have made a substantial, direct and intellectual contribution to the work, and approved it for publication.

## Conflict of Interest

The authors declare that the research was conducted in the absence of any commercial or financial relationships that could be construed as a potential conflict of interest.

## Supplementary Material

Supplementary Figure 1 Prevalence of depression, anxiety, insomnia, work impairment and distress and at-risk categories. PPE: personal Protective Equipment.
