# Supplementary figures and images for: Acute Stress Response Profiles in Health Workers Facing SARS-CoV-2
Source: Front Psychol. 2021 May 31;12:660156. doi: 10.3389/fpsyg.2021.660156 (PMC8201090; doi:10.3389/fpsyg.2021.660156)

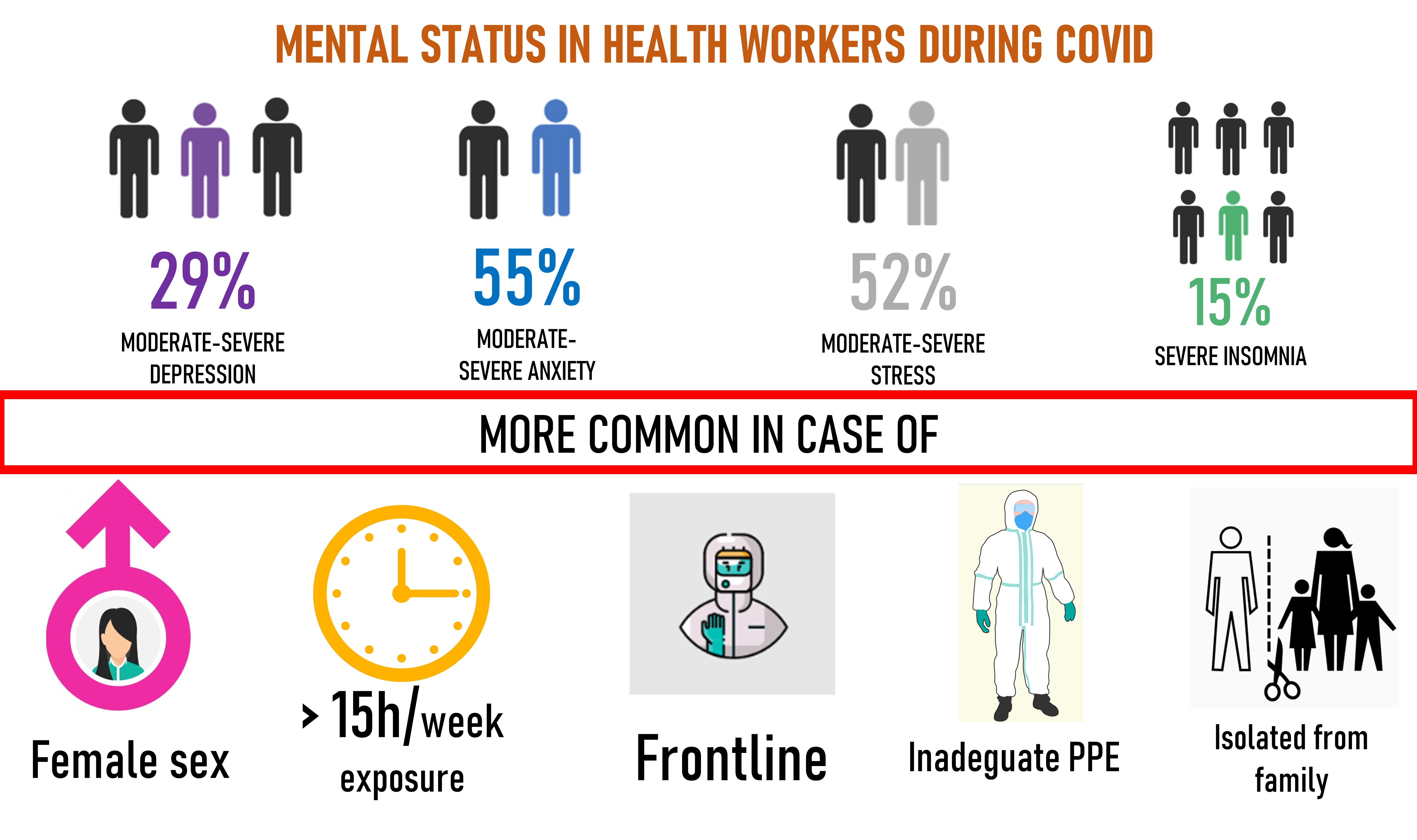

Supplement: Supplementary Figure 1 — Prevalence of depression, anxiety, insomnia, work impairment and distress and at-risk categories. PPE: personal Protective Equipment. [file Image_1.jpg]
